# The effect of aerobic exercise on the number of migraine days, duration and pain intensity in migraine: a systematic literature review and meta-analysis

**DOI:** 10.1186/s10194-019-0961-8

**Published:** 2019-02-14

**Authors:** Joris Lemmens, Joke De Pauw, Timia Van Soom, Sarah Michiels, Jan Versijpt, Eric van Breda, René Castien, Willem De Hertogh

**Affiliations:** 1 Department of Rehabilitation Sciences and Physiotherapy, Faculty of Medicine and Health Sciences, University of Antwerp, Antwerp, Belgium; 2 Department of Otorhinolaryngology, Antwerp University Hospital, Edegem, Belgium; 3 Department of Translational Neurosciences, Faculty of Medicine and Health Sciences, University of Antwerp, Antwerp, Belgium; 4 Department of Neurology, Vrije Universiteit Brussel (VUB), Universitair Ziekenhuis Brussel (UZ Brussel), Laarbeeklaan 101, 1090 Brussels, Belgium; 5 Department of General Practice and Elderly Care Medicine, Amsterdam Public Health research institute, VU University Medical Center, van der Boechorststraat 7, Amsterdam, the Netherlands; 6 Healthcare Center Haarlemmermeer, Waddenweg 1, Hoofddorp, the Netherlands

**Keywords:** Migraine, Headache, Physical therapy, Exercise, Treatment, Headache characteristics

## Abstract

**Background:**

In patients with frequent migraine, prophylactic treatments are used. Patients often request non-pharmacological alternatives. One treatment option can be aerobic exercise. The value of aerobic exercise as prophylactic treatment however needs to be determined.

**Methods:**

A systematic review and meta-analysis was performed to investigate the result of aerobic exercise on the number of migraine days, duration and pain intensity in patients with migraine. After screening three online databases, PubMed, Cochrane library and Web of Science, using predefined in- and exclusion criteria, six studies were retained. Pooling of data was performed when possible.

**Results:**

Significant reductions in the number of migraine days after aerobic exercise treatment were found with a mean reduction of 0.6 ± 0.3 migraine days/month. Other outcomes were too variable to pool due to heterogeneity of outcome measurements. Unpooled data revealed small to moderate reductions in attack duration (20–27%) and pain intensity (20–54%) after aerobic exercise intervention. Various exercise intensities are applied.

**Conclusion:**

There is moderate quality evidence that in patients with migraine aerobic exercise therapy can decrease the number of migraine days. No conclusion for pain intensity or duration of attacks can be drawn. Effect sizes are small due to a lack of uniformity. For future studies, we recommend standardized outcome measures and sufficiently intense training programs.

**Trial registration:**

CRD42018091178.

**Electronic supplementary material:**

The online version of this article (10.1186/s10194-019-0961-8) contains supplementary material, which is available to authorized users.

## Introduction

Worldwide, migraine is the second most disabling disorder [1]. Additionally, in the age group 15–49 years, migraine is the top cause of years lived with disability [1], magnifying its impact on the working population [1]. On average eighteen days per year per migraine patient are missed from work or household activities. Mean annual costs per-person are €1222 for migraine, which leads to high costs for society [2].

The use of a prophylactic treatment is recommended if headache is present more than 8 days per month, disability is present despite acute medication, headache is present more than three days per month when acute medication is not effective [3–6]. These prophylactic drugs, however, might not be tolerated that well by patients or patients might request non-pharmacological alternatives [4, 7, 8]. In migraine, other non-drug related prophylactic treatments like self-management strategies, manual therapy and aerobic exercise are also being employed [9–14]. In aerobic exercise, a moderate intensity training is performed over a longer period of time, e.g. 30 min.

The rationale for using aerobic exercise in migraine is based on the fact that exercise can play a substantial role in the modulation of pain processing [15–18]. Moreover, the analgesic effects of both short-term [16] and long-term [15, 18] aerobic exercise have been observed at both a central and peripheral level [15, 16, 18].

In 2008, the first narrative review on the effect of aerobic exercise in the treatment of migraine showed promising, though inconclusive results [19]. During the past decade, new studies on the use of exercise as a prophylactic treatment in migraine have been published. The updated version of the International Classification of Headache Disorders (ICHD-III) [20] specifically indicates there is a need for a thorough and systematic overview regarding the effects of aerobic exercise in migraine.

Therefore, the aim of the present study is to summarize the literature published after 2004 on the effectiveness of aerobic exercise in migraine. The research question of this systematic review is: what is the effect of aerobic exercise on the number of migraine days, duration and pain intensity in patients with migraine?

## Methods

### Search strategy

The format of this systematic review was based on the Preferred Reporting Items for Systematic reviews and Meta-analyses (PRISMA) [21] (Additional file 1). To establish a search strategy, the PICO format was used [22]. Three electronic databases were searched to identify eligible studies: PubMed, the Cochrane library for trials and Web of Science (from January 1, 2004 till February 21, 2018). An additional search for grey literature was not performed. Inclusion and exclusion criteria were determined as depicted in Table 1. The specific search strategy used for PubMed, the Cochrane library for trials and Web of Science is shown in detail in Table 1 and Additional file 2.

**Table 1:** PICOS and eligibility criteria

	Inclusion criteria	Exclusion criteria
Patients (P)	Migraine with or without aura classified by ICHD-II	Non-human subjects (such as models or animals), other types of headache or pregnant women
Intervention (I)	Physical endurance, physical fitness, aerobic exercise, exercise therapy performed during at least 6 weeks	Manual therapy or medication as stand-alone treatment or no intervention such as diagnosing or performing tests on patients
Control (C)	–	–
Outcome (O)	Number of migraine days, attack frequency, pain intensity or duration of migraine attacks	
Study design (S)	Randomized clinical trials, randomized controlled trials or clinical trial	Non-English, non-Dutch or non-French; studies published before January 1, 2004; cohort studies, case control studies, case reports, reviews or meta-analyses

### Study selection

Based on the predefined inclusion and exclusion criteria the included studies were screened on title and abstract by two investigators (S.M. and J.L.) independently (first screening). Two authors (W.D.H. and J.L.) independently screened the selected full texts (second screening). In case the two authors had diverging opinions, a third author (J.D.P.) was consulted and a decision was made by consensus. Articles were included in the meta-analysis, when data-pooling was feasible based on identical diagnosis (ICHD) and units of outcome measurement.

### Data items and collection

Data were manually extracted from the reports by two researchers (S.M. and J.L.). The reports were searched for the following variables: sample size characteristics (migraine diagnosis); experimental intervention characteristics; exercise intensity; control group characteristics and intervention; follow-up period; results of outcome measures (the number of migraine days, duration of attacks and pain intensity) and confounding factors.

To pool data, the random effect model and RevMan software (version 5.3) was used to compute a mean difference between the data of the intervention and control group. For missing standard deviations the *p*-value or confidence intervals were used to calculate the missing value. These calculations are based on the calculations provided in the Cochrane Handbook [23]. Before entering the mean values in the model, the difference was computed between pre-and post-intervention data of the intervention and control group as it demonstrates the mean reduction in migraine days. A PROSPERO record of this systematic review has been registered (ID: CRD42018091178).

### Risk of bias in the individual studies

**Fig. 1:**
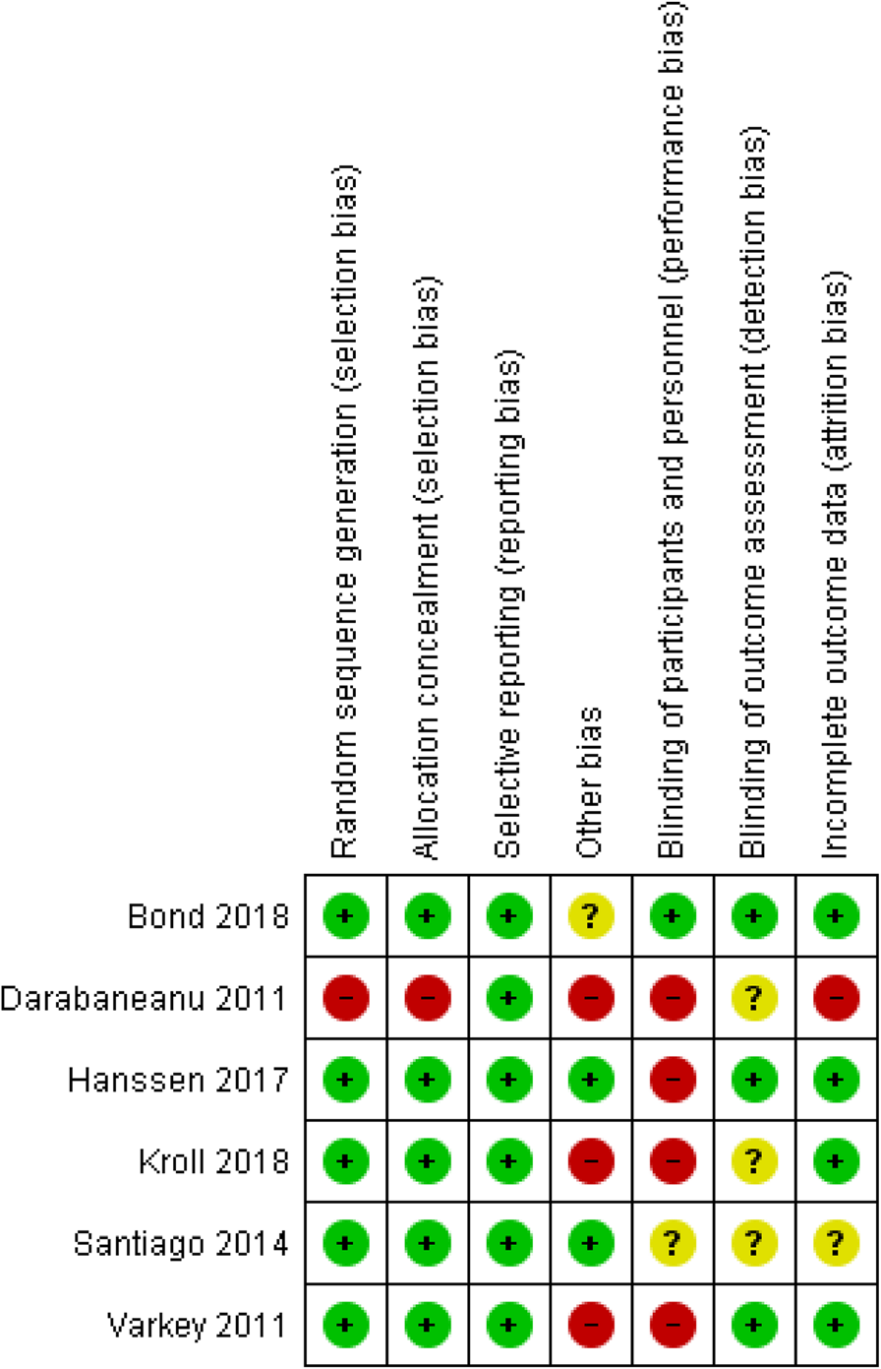
Risk of bias summary: review authors’ judgements about each risk of bias item for each included study (Risk of Bias scale)

**Fig. 2:**
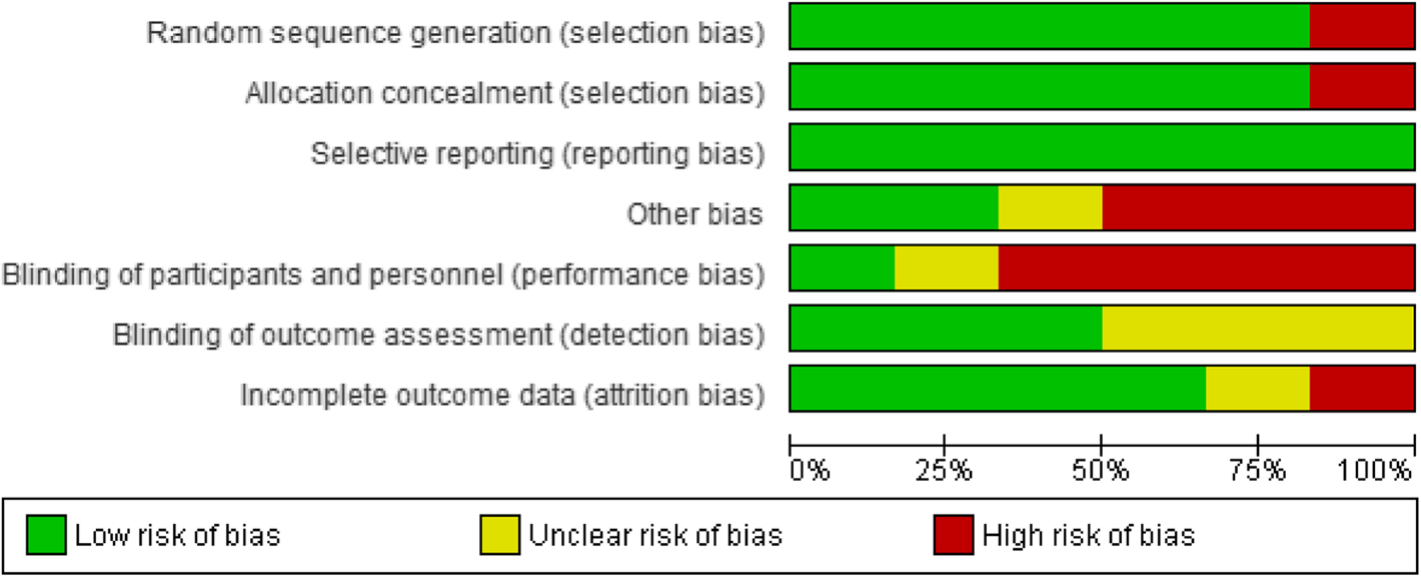
Risk of bias graph: review authors’ judgements about each risk of bias item presented as percentages across all included studies (Risk of Bias scale)

Risk of bias assessment of the selected articles was performed using the Cochrane risk of bias tool (ROB) for randomized controlled trials (RCTs). This checklist can be found in Figs. 1 and 2. Two reviewers (T.V.S. and J.L.) evaluated the included articles independently. The items of the ROB assessment were rated as “1”, “0”, or “?”. An item was rated “1” if sufficient information was available and bias was unlikely. An item was rated “0” if sufficient information was available but the article did not meet a specific criterion. An item was rated “?” if unclear information was provided. Disagreement between researchers was solved by consensus.

Six studies [24–29] were scored using the ROB tool for RCTs. In case of doubt in the analysis of the risk of bias the author of the selected study was contacted. Two authors did not provide additional information.

To measure the level of evidence of each study the classification of the Dutch CBO (Centraal BegeleidingsOrgaan-classificatiesysteem) [30] was used (Table 2).

**Table 2:** Classification of Level of Evidence (Translated from the Dutch classification of CBO)

For articles regarding intervention (prevention or therapy). A1. Meta-analysis containing at least some trials of level A2 and of which the results of individual trials are consistent. A2. Randomized comparative clinical trials of good quality (randomized, double-blind controlled trials) of sufficient size and consistency. B. Randomized controlled trials of moderate (weak) quality or insufficient size or other comparative trials (nonrandomized, cohort studies, patient-control studies) C. Noncomparative trials D. Expert opinions	

## Results

### Study selection

**Fig. 3:**
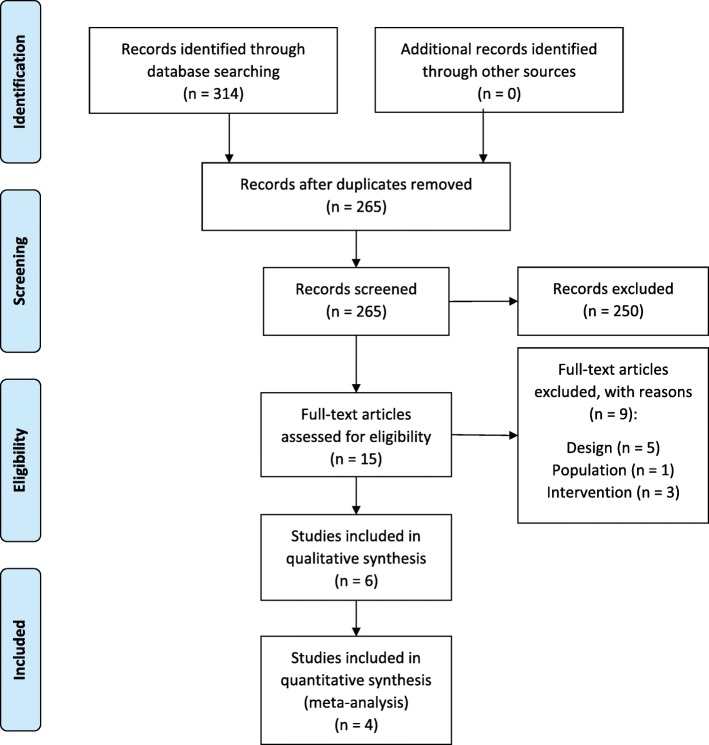
Flow chart of study selection

The search strategy yielded 83 results in PubMed, 53 in the Cochrane library for trials and 194 in Web of Science. After removal of duplicates, 265 articles were screened on title and abstract. Fifteen studies were retrieved and screened on full text by two researchers (W.D.H. and J.L.). After screening on full text, six studies were found eligible and were included in this review (Fig. 3).

### Study characteristics

The included studies were all RCTs, except for one controlled clinical trial (CCT) [25]. All studies included patients with migraine classified by the ICHD-II as mentioned in the inclusion criteria. In three studies, patients were excluded if they performed any kind of regular aerobic training before the start of the study [25] or at least 12 weeks prior to the study [26, 28]. The number of patients enrolled in the different studies ranged from 16 to 110 with a total number of 357 patients with migraine. The mean age of all included patients was 38 years and 88% of them were women. At baseline the mean headache frequency was 9.4 days per month with an average disease duration of 19 years.

### Risk of bias and level of evidence

Overall, a moderate risk of bias was present in all of the included studies. This risk of bias was mostly caused by a high dropout rate and a lack of blinding outcome assessors. In all RCTs, subjects in the control group had similar clinical characteristics as compared to the intervention group at baseline [24–29]. A dropout rate of more than 20% is reported in both intervention and control group in four studies [24, 25, 28, 29]. For this reason item 4 scored negatively in these studies. The design of one study [25] is a non-randomized CCT, therefore item 1 was scored as high risk of bias. All comparative studies [24–29] received a score B according to the CBO [30]. An overview of the risk of bias assessment is presented in Figs. 1 and 2.

### Synthesis of the results

For each individual study, a summary of the characteristics of the participants, type of intervention and main results is presented in Table 3.

**Table 3:** Synthesis of results

Study ID	Patients	Intervention	Intensity	Control	FU	Results	Confounding
Bond 2018 [24]	*N* = 54 MWA/O, ICHD-III ≥ 3 attacks/m 4–20 migraine d/m (3m)	16w BWL program 250min./w 5x/w home-based	Moderate	*N* = 56 Migraine education Self-management	4m	Number of migraine days: / Pain intensity: + 20% Attack duration: + 23% All results: NS^a^	Overweight or obese (BMI = 25–49.9 kg/m^2^) Preventive/abortive pharmacological treatment if stable regimen ≥2m
Darabaneanu 2011 [25]	N = 8 MWA/O, ICHD-II ≥ 2 attacks/m Prior: No aerobic training	10w jogging 50min. 3x/w supervised 1/3 @ home	60–75% VO_2_peak	*N* = 8 No intervention	8w	Number of migraine days: − 39% Pain intensity: − 20% Attack duration: − 20%	Dropout 50%
Hanssen 2017 [26]	*N* = 30 I1 = 15 (HIT) I2 = 15 (MCT) EM without aura, ICHD-IIIb Prior: No regular exercise No prophylaxis 8w	12w HIT (4 times) 2x/w 4min. 90% 3min. rest 70% 12w MCT, 2x/w 45min. 2x/w supervised	HIT: 90–95% HR MCT: 70% HR	*N* = 15 Maintain daily physical activity and physical activity recommendations	/	Number of migraine days: −29% (MCT) − 63% (HIT) Pain intensity: / Attack duration: / All results: NS^a^	
Krøll 2018 [29]	*N* = 36 EM and CM combined with NP and TTH, ICHD-IIIb ≥ 2 attacks/m	3m cycling/cross-training/brisk walking/running 3x/w 45min. 1x/w supervised 2/3 @ home/gym	RPE scale 14–16	N = 36 Maintain daily physical activity	3m	Number of migraine days: −22% Pain intensity: − 20% Attack duration: − 23%	Participants engaged in some form of exercise activity could continue. Preventive and acute medication allowed.
Santiago 2014 [27]	*N* = 24 CM, ICHD-II Prior: No exercise for 3m No prophylaxis	12w fast walking + amitriptyline (25mg/d) 3x/w 40min. supervised weekly by telephone	Aerobic (HR + Borg)	*N* = 26 25mg/d amitriptyline	12w	Number of migraine days: − 78% Pain intensity: − 54% Attack duration: − 27%	Amitriptyline use (TCA)
Varkey 2011 [28]	*N* = 16 MWA/O, ICHD-II 2–8 attacks/m > 1y migraine ^b^ before age of 50 Prior: < 1x/w exercise 12w	12w indoor cycling 3x/w 40min. supervised ≥ 2/3 @home	RPE scale 14–16	*N* = 31 Relaxation (*N* = 14) 5-20min./w Topiramate (*N* = 17) 25mg/w - 200mg/d	10-12m	Number of migraine days: −28% Pain intensity: − 18% Attack duration: / All results: NS^a^	50% of all ITT patients have 6m FU

Legend: ^a^ between-group differences, ^b^ onset, *BWL* behavioral weight loss program, *C* control group, *CM* chronic migraine, *d* day(s), *FU* follow-up, *HIT* high-intensity interval training, *HR* heartrate, *I* intervention, *ICHD* international classification of headache disorders, *ITT* intention-to-treat analysis, *m* month(s), *MCT* moderate continuous aerobic training, *MWA/O* migraine with/without aura, *N* number of, *NP* neck pain, *NS* non-significant, *PP* per-protocol analysis, *RPE* rate of perceived exertion, *TCA* tricyclic antidepressant, *TTH* tension-type headache, *w* week(s)

#### Interventions

Several types of aerobic exercise were used in the studies. One study used a walking program [27], one a combination of cross-training, walking, jogging and cycling [29], two a jogging protocol [25, 26], one a behavioral weight loss program [24] and finally cycling was used in one study [28]. The walking program [27] consisted of 40–45 min of fast walking and was controlled by heartrate and Borg-scale or Rate of Perceived Exertion scale (RPE) [31]. The patients also received 25mg amitriptyline each day [27].

Jogging was performed using [1] an interval program [26] (jogging and walking) or [2] a continuous run of moderate intensity for 30–45 min [25, 26]. To assure patients trained in the aerobic zone (the zone where oxygen is adequately available for the energy production process), heart rate or RPE was measured during warm up, exercise and the cooling-down period.

Indoor cycling training consisted of 15 min warming up, 20 min exercising at moderate intensity and 5 min cooling down using percentages of VO_2_peak and Borg-scale or RPE [28].

One study [29] used a combined protocol of cross-training, brisk walking, running or indoor cycling. This training protocol comprised 10 min warming up, 30 min exercising and 5 min cooling down, using RPE to ensure aerobic training [29].

The behavioral weight loss program was designed to accomplish a ≥ 7% weight loss goal in sixteen weeks. In order to achieve this goal, participants performed a gradually progressed exercise protocol to a goal of 250 min per week, a standard calorie- and fat-restricted diet, home-based exercise (50 min, 5 days/week) and were provided instructions in behavioral modification strategies [24].

All participants in the intervention groups trained at least 3 times per week, except in one study [26]. In three studies patients were instructed to train at the local gym, at a maximum frequency of twice per week, if they could not attend the supervised training sessions [25, 28, 29]. To evaluate if patients were training in the aerobic zone, heart rate [25–27], Borg-scale or RPE [26–29] and percentages of VO_2_peak [26, 28] were monitored. In one study the training intensity was not monitored [24].

#### Outcome

Patients kept diaries to report on the the number of migraine days, attack duration, pain intensity and the use of analgesic medication. The reported outcomes were computed from these diaries. Assessments were performed before, during and after the aerobic exercise treatment. The total follow-up period ranged from 8 weeks to 12 months. In one study no follow-up period was used [26].

#### Controls

Six studies compared the results of the intervention group with randomized control groups [24–29], only one study had an age-and gender-matched control group [25]. Patients with migraine included in the control groups received either no intervention [25], a treatment based on medication (25mg amitriptyline/day) [27], education [24], advice to maintain a habitual daily activity profile [26, 29], relaxation therapy or topiramate (25mg/week - max. 200mg/day) [28]. In comparison to topiramate treatment, aerobic exercise and relaxation therapy were found to be equally effective regarding the attack frequency and the number of migraine days [28]. Concerning pain intensity, a greater reduction was reported favoring the topiramate group (37%) compared to aerobic exercise (10%) and relaxation therapy (9%) [28]. Moreover, combining amitriptyline and aerobic exercise had a significant effect on the number of migraine days, pain intensity and attack duration compared to amitriptyline treatment alone [27]. In comparison to maintaining daily physical activity and moderate continuous training, high intensity interval training showed larger, although statistically not significant, effect sizes for decreasing the number of migraine days per month [26]. Migraine education and self-management showed an equal effect on pain intensity and attack duration compared to a behavioral weight loss program [24].

#### Effect of aerobic exercise on the number of migraine days

**Fig. 4:**
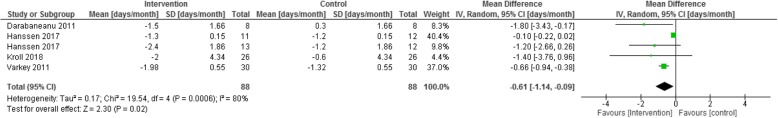
Pooled data comparing intervention and control group on the number of migraine days (days/month)

Three out of six studies reported a significant reduction in the number of migraine days ranging from 22% to 78% [25, 27, 29]. Data-pooling of four studies [25, 26, 28, 29], with a total of 176 patients, show a significant effect of aerobic exercise on the number of migraine days at 10–12 weeks (*p* = 0.0006). A mean reduction of 0.6 ± 0.3 migraine days/month was found favoring the intervention group (Fig. 4). These studies were pooled based on similar diagnosis of migraine, intervention and outcome. Hanssen et al. [26] was mentioned twice as both moderate continuous aerobic training and high-intensity interval training were compared to the control group.

#### Effect of aerobic exercise on pain intensity and attack duration

Three studies [25, 27, 29] reported a reduction of 20% up to 54% in pain intensity after aerobic exercise combined with a decrease in attack duration of 20–27%. These outcomes (pain intensity and duration) were not pooled due to the heterogeneity of the used units of outcome measurement. For instance duration of attacks was measured in hours per attack [29], hours per month [25] or in different time intervals (6h–12h-18h-24h) [27]. Additionally, Varkey et al. [28] showed a decrease in the use of analgesic medication (71%) in the topiramate group, 6 months after treatment according to the per-protocol analysis. This result was not found in the intention-to-treat analysis. Two other studies [27, 29] measured and reported acute medication use, but no significant between-group differences were found. The first study found no significant differences in acute medication use when comparing a pharmacological treatment to a combined pharmacological and exercise treatment [27]. In the second study, acute medication use decreased non-significantly in the exercise group compared to a group maintaining normal daily activity [29].

## Discussion

The aim of this systematic review was to explore the effect of aerobic exercise in patients with migraine on the number of migraine days, attack duration and pain intensity. Moderate quality evidence indicates that in patients with migraine aerobic exercise therapy decreases the number of migraine days. Low quality evidence indicates that aerobic exercise can decrease pain intensity or duration of migraine attacks. To our knowledge, the only other existing review on this topic was published in 2008 [19]. However, Busch et al. [19] acknowledged themselves that none of the included studies in this narrative review met valid criteria of good clinical practice. Therefore, a systematic review was conducted to explore the effects of aerobic exercise using higher quality studies.

Five RCTs [24, 26–29] and one CCT [25] published after 2004, reporting on the effect of aerobic exercise in patients with migraine, were included in this review. The risk of bias of the included trials was low to moderate with a high risk of performance and detection bias due to a lack of blinding of participants, personnel and outcome assessors.

Based on our meta-analysis, there is moderate evidence that aerobic exercise can lead to a decrease of 0.6 migraine days per month. The clinical relevance of this finding is low. However, it may be of interest if it is added to the value of current usual care. Furthermore, higher training intensities might provide interesting results as the training intensity in the included studies was low. This finding is in line with the findings of Busch et al. [19], who found a decrease of 3.7 migraine days per month. However, this result is based on a single report. In their review two RCTs [32, 33] and six single cohort studies [34–38] were included. However, as mentioned above none of those studies met valid criteria of good clinical practice [19]. In 2015, Luedtke et al. [39] evaluated interventions used by physiotherapists for patients with headache, such as aerobic exercise. Based on six studies, of which the data of one study was not estimable, their meta-analysis indicated a reduction of 2.99 days with migraine, although not significant (*p* = 0.23). In contrast, pooling of data from one CCT [25] and three RCTs [26, 28, 29] in this review shows a significant reduction of migraine days per month. We obtained the mean reduction by using the difference between pre- and post-intervention data. Additionally, all studies provided a long-term exercise protocol for at least ten weeks. This can explain the difference between our results and those in the systematic review of Luedtke et al. [39].

Interestingly, we found that topiramate and tricyclic antidepressants show similar results compared to aerobic exercise in decreasing the number of migraine days per month [28]. Aerobic exercise appears to be a valuable alternative, taking into account that side effects are common with a pharmacological treatment, such as weight changes, memory loss and fatigue [3, 40, 41].

Regarding duration of migraine attacks small to moderate reductions (20–27%) were reported [25, 27, 29], such as a reduction of 20 migraine hours post-treatment in one study [29]. This result is similar to the conclusions of Busch et al. [19]. Due to the heterogeneity of the units of the outcome measurement, interpreting raw data was difficult.

The results of the present review suggest that aerobic exercise can reduce pain intensity (20–54%) in patients with migraine [25, 27, 29], confirming the findings of Busch et al. [19]. The analgesic effects on central and peripheral levels have already been reported [15, 16, 18] but the heterogeneity of the units of the outcome measurement might have biased the results.

Additionally, there is low quality evidence that patients use less analgesic medication as an effect of aerobic exercise [28]. These results contradict the findings of Busch et al. [19], who concluded that analgesic medication intake was not altered by aerobic exercise.

Our review shows low quality evidence for greater treatment effects by combining aerobic exercise with amitriptyline [27].

While our review focuses on the influence of aerobic exercise on clinical parameters of migraine, its underlying mechanisms were beyond the scope of our review. Other reviews provide some hypotheses regarding these mechanisms [9, 11, 42, 43].

This review’s patient population consisted of 88% females and 12% males. This is an expected distribution, as a 3:1 female:male ratio is reported in other epidemiologic studies [44]. In the current review, the inclusion criteria were: patients with migraine with and without aura according to the ICHD-II. A similar diagnosis is a major strength of this review as it ensures a homogeneous group and allows pooling of data. Additionally, in all studies patients with and without aura were included. Therefore, patients can easily be compared between studies. However, the control groups consisted of usual care treatments (topiramate and amitriptyline) [27, 28], alternative treatments (relaxation, maintain daily physical activity and migraine education) [24, 26, 28, 29] and no treatment [25]. This may have influenced the comparability, since there might be differences between control groups that received treatment (active controls) and control groups that received no treatment at all (passive controls). Interestingly, no significant difference is found if active controls are compared to aerobic exercise (topiramate, relaxation, migraine education and maintaining habitual function with standard physical activity recommendations) [24, 26, 28]. One can state that these active groups are equally effective compared to aerobic exercise. Significant treatment effects are found, when comparing aerobic exercise with no treatment or maintaining habitual function [25, 29].

Dropout rate in total was high in four of the included studies, respectively 28% [29], 33% [24] and 50% [25, 28]. The most important reason for withdrawal of participants was lack of time to get to and attend three supervised exercise training sessions per week. Since stress is an important trigger for migraine attacks, Varkey et al. [45] suggested home-based training programs to improve compliance and to reduce stress levels [46]. On the other hand, home-based training might be less therapy compliant, which could lead to false interpretation. Positive findings have been suggested for supervised home-based programs [19, 35, 45], although these last two showed a high risk of bias due to the lack of a control group and subjective endpoints.

Our review population is mainly comprised of untrained patients with migraine. This selection of subjects might have biased the results as this does not necessarily represent a typical migraine population [19]. A moderate intensity level training was chosen to avoid exercise-induced migraine and other negative side effects [28, 29]. Aerobic training was recommended by the American College of Sport Medicine (ACSM) [47] as training 3–5 days a week, 20–60 min, with an intensity of 55/65–90% of maximum heart rate. In this review patients exercised according to the ACSM recommendations of aerobic training for a period of 10 weeks or more with moderate intensity [47]. Positive findings were measured in the intervention group and no negative side effects were registered in any of the trials. Larger exercise volumes, such as high-intensity training or higher exercise duration, seem to be related to larger reductions in the number of migraine days in the intervention group [25, 26, 29].

### Recommendations for further research

Major gaps exist in the current knowledge on the effect of aerobic exercise on patients with migraine. Further research to study the effects reported in this systematic review are mandatory to unravel the mechanisms of physical training on migraine [11, 42]. We recommend that future studies use uniform outcome measures of headache characteristics as recommended by the International Headache Society [48], use blinded assessors, provide homogeneous patient samples, design randomized controlled trials comparing aerobic training in patients with migraine with and without supervision to explore the difference between both protocol types, investigate the effect of larger exercise volumes as an intervention protocol and finally investigate the combined effect of pharmacological treatment and aerobic exercise in comparison to a pharmacological treatment alone.

## Conclusion

Based on the results of this review, there is a moderate evidence that aerobic exercise decreases the the number of migraine days [25, 26, 28, 29]. Additionally, there is low quality evidence that aerobic exercise decreases the attack duration and pain intensity [25, 27, 29].

## Additional files

Additional file 1: PRISMA checklist. (DOCX 17 kb)

Additional file 2: Search string. (DOCX 12 kb)
