# Supplementary material for: The effect of aerobic exercise on the number of migraine days, duration and pain intensity in migraine: a systematic literature review and meta-analysis
Source: J Headache Pain. 2019 Feb 14;20(1):16. doi: 10.1186/s10194-019-0961-8 (PMC6734345; doi:10.1186/s10194-019-0961-8)
Supplement: Supplementary file 2 — Search string. (DOCX 12 kb) [file 10194_2019_961_MOESM2_ESM.docx]

Appendix 2: Search string

**PubMed**

("Migraine Disorders"[MeSH Terms] OR "Migraine Disorders"[All Fields] OR "Migraine"[All Fields]) AND ("Physical Endurance"[MeSH Terms] OR "Physical Fitness"[MeSH Terms] OR "Exercise"[MeSH Terms] OR "Exercise Therapy"[MeSH Terms] OR Aerobic[All Fields])

Filters:

- From 1/01/2004 till 21/02/2018
- Language: English, French and Dutch

**Web of Science**

TS=(("Migraine Disorders" OR "Migraine") AND ("Physical Endurance" OR "Physical Fitness" OR "Exercise" OR "Exercise Therapy" OR “Aerobic”))

Filters:

- From 1/01/2004 till 21/02/2018
- Language: English, French and Dutch

**The Cochrane library for trials**

(("Migraine Disorders" OR "Migraine") AND ("Physical Endurance" OR "Physical Fitness" OR "Exercise" OR "Exercise Therapy" OR “Aerobic”))

Filters:

- From 1/01/2004 till 21/02/2018
